# Molecular embedding-based algorithm selection in protein-ligand docking

**DOI:** 10.1186/s13321-026-01168-8

**Published:** 2026-03-14

**Authors:** Jiabao Brad Wang, Siyuan Cao, Hongxuan Wu, Yiliang Yuan, Mustafa Mısır

**Affiliations:** 1 Division of Natural and Applied Sciences, Duke Kunshan University, 8 Duke Av., Suzhou, 215316 Jiangsu China; 2 Machine Learning Department, Mohamed bin Zayed University of Artificial Intelligence, Building 1B, Masdar City, Abu Dhabi UAE

**Keywords:** Algorithm selection, Cheminformatics, Molecular embeddings, Pose evaluation, Protein-ligand docking, Docking benchmarks

## Abstract

Selecting an effective docking algorithm is highly context-dependent, and no single method performs reliably across structural, chemical, and protocol regimes. MolAS is a lightweight algorithm-selection model that predicts per-algorithm performance from pretrained protein and ligand embeddings using attentional pooling and a shallow residual decoder. With hundreds to a few thousand labelled complexes, MolAS achieves up to a 15 percentage-point absolute improvement over the single best solver (SBS) and closes 17–66% of the virtual best solver (VBS)–SBS gap across five docking benchmarks. Analyses of selection frequencies, margin-conditioned reliability, and benchmark-level oracle structure indicate that MolAS is most effective when the workflow-defined oracle landscape has low winner entropy and a reasonably separable top-solver region, but degrades under protocol mismatch that shifts solver rankings and changes the induced labels. These results suggest that, in the evaluated regime, robustness is limited less by representational capacity than by workflow- and protocol-induced instability in solver hierarchies, positioning MolAS as an in-domain selector for fixed pipelines and as a diagnostic tool for assessing when docking algorithm selection is well-posed.

**Scientific Contribution**: MolAS introduces a controlled, embedding-based selector that reduces dependence on heavy graph encoders, enabling a cleaner separation between representational choices and workflow-defined label structure. A cross-benchmark and cross-protocol analysis links selection success and failure to oracle entropy, near-ties among top solvers, and protocol-induced ranking shifts, providing an evidence-backed diagnostic account of when docking algorithm selection is likely to yield gains. The findings differentiate this work from prior docking AS studies that report in-domain improvements under a single fixed workflow by explicitly characterising protocol dependence and motivating protocol-aware modelling as a route to stronger generalisation.

**Graphical Abstract:**

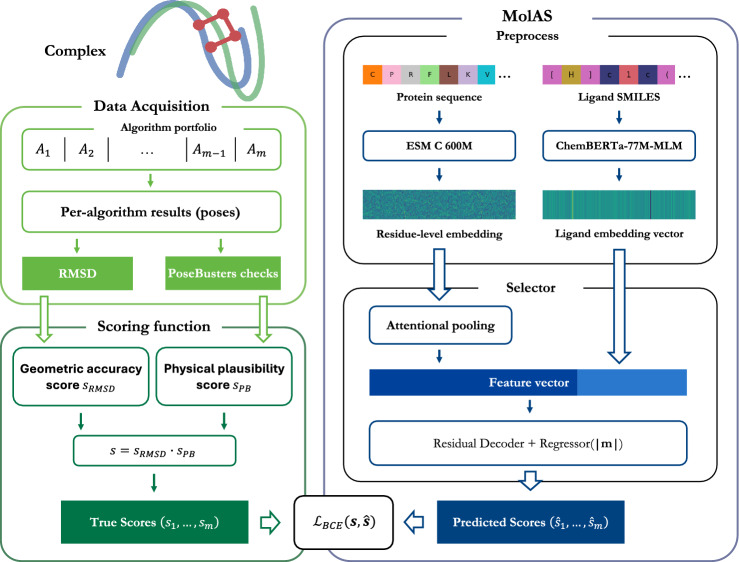

**Supplementary Information:**

The online version contains supplementary material available at 10.1186/s13321-026-01168-8.

## Introduction

Molecular docking is the computational process that predicts the binding configuration of a ligand to its target protein at atomic resolution and is central to structure-based drug discovery [1]. Traditional docking pipelines rely on empirical scoring functions coupled with heuristic search [2–4], while classical scoring functions have historically spanned physics-based, empirical, and knowledge-based formulations [5–7]. These methods balance speed and physical interpretability, but their docking scores often show limited correlation with experimentally measured binding affinities or binding constants [8]. Recent machine learning (ML)-based models such as DiffDock [9] and SurfDock [10] aim to improve pose generation or scoring by learning protein-ligand interaction patterns directly from data. Despite decades of development, no single method consistently excels across all docking scenarios, reflecting both the *No Free Lunch Theorem* [11] and the profound context dependence of protein-ligand recognition [12].

Algorithm Selection (AS) [13] offers a principled route to adaptivity in such heterogeneous regimes. Rather than committing to a single solver or fixed configuration, AS systems learn to recommend the algorithm predicted to perform best for each instance [14]. Beyond docking, AS has been operationalised at scale in AutoML systems such as Auto-WEKA [15] and Auto-sklearn [16], and has shown consistent benefits in molecular modelling tasks such as QSAR regression via meta-learning [17].

In docking, initial AS studies focused on *within-engine* choice. Chen et al. [18] proposed the first docking-specific AS framework using the *ALORS* recommender [19] to select among AutoDock configurations from tabular descriptors, and subsequent work explored related regression-based parameter tuning [20]. More recent efforts extend AS to *cross-algorithm* selection across multiple docking engines, using molecular descriptors or learned representations to predict per-solver performance [12, 21–23]. These approaches demonstrate that instance-wise solver choice can outperform a single best solver (SBS) in-domain, but they are typically evaluated under a fixed benchmark and protocol, and often rely on relatively heavy graph-based encoders.

This combination leaves an important ambiguity unresolved: observed gains can reflect genuine improvements in molecular representation, but can also arise from benchmark- and workflow-specific effects, such as protocol-induced shifts in solver rankings and instability of the empirical oracle used to define training labels. When both the representation and the workflow vary implicitly, it is difficult to separate representational benefit from distributional or procedural alignment. Table 1 summarises representative docking AS formulations and highlights which factors are controlled (representation, objective, and protocol/workflow consistency).

**Table 1:** High-level comparison of MolAS with representative docking algorithm-/protocol-selection approaches, focusing on what is controlled in the formulation and evaluation

**Method**	**Selection scope**	**Input representation**	**Learning objectives**	**Protocol variation evaluated**
ALORS-based config selection [18]	within-engine	Tabular ligand features (RDKit descriptors + PubChem substructure fingerprints)	Recommender-style selection/ranking of configurations (ALORS)	Fixed benchmark/protocol with multiple within-engine configurations
Parameter/protocol tuning [20]	within-engine	Tabular ligand features (molecular descriptors + substructure fingerprints)	predicts docking scores with MAE/MSE/RMSE	Fixed benchmark/protocol with multiple within-engine configurations
Cross-algorithm selection (descriptors) [12]	cross-engine	Tabular protein statistics + ligand RDKit descriptors	Predicts RMSD (squared loss)	Fixed benchmark/protocol and portfolio
GNNAS-Dock [23]	cross-engine	Residue-level protein graph + atom-level ligand graph (GNN features; stacking/meta-model)	Accuracy model predicts RMSD prediction; Efficiency model predicts binary success (RMSD $<2$ Å) + runtime	Fixed benchmark/protocol and portfolio
MC-GNNAS-Dock [21]	cross-engine	Residue-level protein graph (pretrained node embedding) + atom-level ligand graph (physicochemical node embedding)	Predicts PoseBuster-validity-gated RMSD score with BCE loss; ranking loss tested	Fixed benchmark/protocol and portfolio
MolAS (this work)	cross-engine	Residue-level pretrained protein node embedding + pretrained ligand embedding	Predicts PoseBuster-validity-gated RMSD score with BCE loss; ranking loss tested	Multiple benchmarks & post-processing

The present work introduces MolAS (Molecular Embedding–Based Algorithm Selector), a lightweight selector designed to probe this ambiguity. MolAS replaces graph encoders with pretrained molecular language model embeddings for proteins (ESM C [24]) and ligands (ChemBERTa [25]), coupled with a minimal attentional pooler and shallow residual decoder. This design aims to isolate the role of molecular representation from that of workflow-defined oracle landscapes, enabling a more direct assessment of whether docking AS is primarily constrained by representational capacity or by data and protocol variability.

We evaluate MolAS on a curated BindingMOAD dataset and across three modern docking benchmarks spanning diverse structural regimes and pose-generation protocols. In most in-domain settings, MolAS outperforms the single best solver (SBS) and closes a substantial portion of the virtual best solver (VBS)–SBS gap with hundreds to a few thousand labelled complexes, depending on the benchmark. However, performance deteriorates sharply when training and test protocols differ, indicating that MolAS inherits the oracle hierarchy of the workflow on which it is trained. This highlights a practical constraint of docking AS: changes in dataset curation, docking engines, or post-processing can effectively redefine the selection problem.

Ablation studies show that neither deeper encoders nor alternative objective functions provide systematic improvements, reinforcing that solver-hierarchy instability, benchmark-specific oracle entropy, and protocol-induced shifts in ranking dominate performance. Overall, MolAS is positioned as a workflow-adaptive selector under fixed docking protocols and as a diagnostic tool that clarifies when docking AS is feasible (non-trivial VBS–SBS gap and separable solver regimes) versus when an SBS-like policy is a reasonable default. These findings suggest that robust cross-protocol AS will require explicit modelling of workflow changes rather than further architectural scaling.

In summary, the contributions are threefold. Methodologically, MolAS provides a deliberately lightweight, embedding-based selector that uses pretrained protein and ligand language model representations with a minimal pooling and decoding head, so that selection performance can be studied without conflating it with increasingly complex graph encoders. Empirically, MolAS is evaluated across a curated BindingMOAD split and multiple modern docking benchmarks spanning distinct protocols and post-processing settings, including systematic ablations that test whether heavier encoders or alternative objectives yield consistent gains. Conceptually, the study frames docking algorithm selection as a *workflow-defined* problem: solver hierarchies and training labels are shaped by dataset curation and pipeline choices, and cross-protocol shifts and oracle instability can dominate apparent progress, motivating MolAS both as a workflow-adaptive selector under fixed protocols and as a diagnostic tool for assessing when selection is well-posed.

## Materials and methods

### Pipeline overview

MolAS is an algorithm selection (AS) system. Let $\mathcal {X}$ denote a class of problem instances and $\mathcal {A} = \{A_1, \dots , A_m\}$ a finite set of *m* candidate algorithms. An AS system is a mapping

1 $$\begin{aligned} S : \mathcal {X} \rightarrow \mathcal {A} \end{aligned}$$

that selects, for each $\textbf{x}\in \mathcal {X}$, an algorithm expected to perform well under a task-specific performance measure $\phi : \mathcal {A} \times \mathcal {X} \rightarrow \mathbb {R}_{\ge 0}$ [13]. In practice, $\phi$ is unknown and approximated by a learned model $\hat{\phi }: \mathcal {A} \times \mathcal {X} \rightarrow \mathbb {R}_{\ge 0}$. The system then defines

2 $$\begin{aligned} S(x) := \arg \max _{A \in \mathcal {A}} \hat{\phi }(A, \textbf{x}), \end{aligned}$$

thereby inducing selection via performance prediction. Often, it is modelled $(\hat{\phi }(A_1, \textbf{x}),..., \hat{\phi }(A_m, \textbf{x})) = \hat{\boldsymbol{\phi }}(\textbf{x}) = g(f(\textbf{x}))$ for a feature extractor (encoder) $f: \mathcal {X} \rightarrow \mathbb {R}^d$ and performance predictor (decoder) $g:\mathbb {R}^d \rightarrow \mathbb {R}^m$ learned to reconstruct the performance/score labels $\textbf{s}\in \mathcal {S}$, yielding the composition

3 $$\begin{aligned} S = \arg \max \circ g \circ f. \end{aligned}$$

**Fig. 1:**
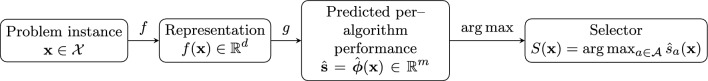
Schematic of the algorithm selection mapping in the sense of [13]. Each instance $\textbf{x}$ is mapped to a feature vector $f(\textbf{x})$, from which a predictor *g* produces a vector $\hat{\textbf{s}}$ of estimated performances for all algorithms in the portfolio $\mathcal {A}$. The selector chooses the algorithm with maximal predicted performance

Such a system, as shown in Fig. 1, induces a total ranking over $\mathcal {A}$ for each problem instance by its characteristics, with selection yielding the top-ranked algorithm. In our work, each $\textbf{x}= (\textbf{x}_P, \textbf{x}_L)$ represents the input pair constructed from a protein-ligand pair ("Preprocessing" section), *f*, *g* are learned by an attentional pooler and a residual multilayer perceptron (MLP) decoder respectively ("Model architecture" section), and the target performance metrics are formulated with a physics-inspired composite scoring function ("Scoring function" section).

**Table 2:** Candidate docking algorithms for algorithm selection (on *MOAD-curated*)

Method	Type	Central mechanism
Smina [3]	Classical	Vina with empirical scoring and local search
Qvina-W [2]	Classical	Vina with fast stochastic global optimisation
DiffDock [9]	ML-based	Diffusion model for direct pose generation
DiffDockL [26]	ML-based	DiffDock with flexible ligand modeling
SurfDock [10]	ML-based	*SE*(3)-equivariant diffusion GNN
Gnina [27]	ML-based	3D CNN-based rescoring of docking poses
Uni-Mol Docking V2 [28]	ML-based	Transformer on atomic coordinates with pretraining
KarmaDock [29]	ML-based	*SE*(3)-equivariant attention

### Datasets and preprocessing

#### Dataset

A proper AS dataset involves problem instances (protein–ligand pairs) $\mathcal {X} = \{\textbf{x}_1,\ldots ,\textbf{x}_n\}$ and score targets $\mathcal {S}\in \mathbb {R}^{n\times m}$ that contain per-instance, per-algorithm performance data. Acquiring such data is inherently challenging: candidate docking pipelines are heterogeneous, their training data may overlap with common benchmarks, and exhaustive evaluation over large instance sets is costly. This necessitates a strategic selection of both representative molecular instances and a portfolio that is (i) methodologically diverse and (ii) available under consistent, documented protocols.

Accordingly, the main AS experiments use a curated portfolio of eight docking algorithms, chosen to span strong baselines and learning-based approaches while remaining tractable for systematic analysis across workflows (Table 2). Portfolio completeness is not claimed; rather, this set aims to analyse algorithm selection under a representative and protocol-consistent set of solvers.

Since most of these algorithms have parsed *PDBBind* [30] as the training set, we evaluate them on *BindingMOAD* [31], a comprehensive dataset of experimentally determined protein–ligand complexes. To reduce potential overlap with common training corpora and to obtain a chemically standardised evaluation split, a *MOAD-curated* subset is constructed from BindingMOAD using a fixed, two-stage filtering procedure that does *not* consult docking scores, solver rankings, RMSD, or pose validity at any point.

First, starting from BindingMOAD, a working pool of approximately 6700 complexes is formed by removing complexes whose PDB IDs appear in the PDBBind v2020 collection and by excluding overlaps with the DockGen protein lists, followed by basic dataset housekeeping (e.g., resolving duplicates and incomplete entries). Second, the working pool is filtered using fixed protein and ligand quality criteria implemented in a standalone script: on the protein side, only structures composed of the 20 canonical amino acids are retained; on the ligand side, RDKit-based checks enforce standard Lipinski-style constraints (molecular weight $\le 500~\textrm{Da}$, $\log P \le 5$, $\le 5$ hydrogen bond donors, and $\le 10$ hydrogen bond acceptors). Complexes failing any criterion are removed. The surviving 3179 complexes constitute the final *MOAD-curated* dataset used in the main experiments.

Solver performance is analysed post hoc on this fixed dataset to characterise solver dominance and the resulting VBS–SBS gap. Each algorithm demonstrates strong performance in certain docking scenarios, ensuring the presence of a non-trivial selection problem. In addition, complementary analyses use extra benchmarks that provide released per-complex outputs for a substantially broader catalogue of docking and co-folding pipelines (including recent methods such as Interformer) under documented settings, which we describe next.

#### Extra benchmarks

In addition to MOAD-curated, two external benchmarks, *PoseX* [32] and *PoseBusters* [33] are included to assess the model’s performance under distinct data distributions.

*PoseX*

*PoseX* is a docking benchmark including 718 self-docking cases (*PoseX-SD*) and 1312 cross-docking cases (*PoseX-CD*). Self-docking refers to docking a ligand back into the same protein conformation from which it was crystallised and is the appropriate analogue of standard *redocking*-based docking-power evaluation. Cross-docking docks each ligand into non-cognate conformations of the same protein, introducing receptor mismatch (backbone and side-chain differences) that better reflects scenarios where the exact receptor structure is unavailable; it is therefore treated here as a robustness regime rather than a definition of docking power. These cases, alongside another benchmark dataset of 85 complexes, the *Astex Diverse Set* [34], are evaluated across 24 algorithms. These methods span physics-based docking like AutoDock Vina, machine learning-based docking models such as DiffDock and SurfDock, and AI co-folding frameworks that can implicitly orient ligands by joint protein-ligand structure prediction, e.g. AlphaFold 3. Each algorithm is treated as an end-to-end docking pipeline executed under its recommended/default settings, including any method-specific preparation, repair, or physics-based optimisation refinement steps used to produce the final pose, as documented in [32]. These default settings include method-specific search and sampling budgets (e.g., number of generated poses/samples and method-internal ranking procedures), which can materially affect docking quality and therefore shape the induced solver-performance landscape on which algorithm selection is defined. PoseX evaluates the *top-1 ranked* pose returned by each pipeline, and documents per-method inference/sampling settings; accordingly, sampling budget is treated here as part of the solver protocol definition rather than a controlled variable. Standardising budgets across methods could change solver rankings and is naturally subsumed under protocol-aware selection, rather than the present out-of-the-box pipeline selection setting.

PoseX additionally provides an optional post-processing step termed relaxation, intended to correct steric clashes and strained local geometries by locally adjusting atom positions [32]. To separate the effect of this universal wrapper from solver-specific processing, results are reported both on the native pipeline outputs (*no post-processing*) and after applying relaxation once to each pipeline’s output pose; the associated changes in docking RMSD and PoseBusters-validity are summarised in Appendix A.1. Overall, PoseX yields a benchmark that captures both near-native and cross-conformation docking scenarios while retaining comparability across diverse algorithm families.

*PoseBusters*

 The *PoseBusters* (V2) benchmark provides a comprehensive validation framework for ligand poses. Its focus extends beyond RMSD accuracy, prioritizing the physical plausibility of docking outputs. It contains 428 complexes and provides predictions from 7 modern docking models. Alongside with the standard output poses, a unified molecular-mechanics energy minimisation post-processing step is applied to generate a mirror group of refined poses. During this process, the ligand is optimised with OpenMM using the AMBER ff14SB (protein) and SMIRNOFF/Sage (ligand) force fields while the receptor is held fixed, producing an MM-minimised (MM-min) pose for standardised comparison [33].

We note that several widely used datasets in molecular modelling, such as CASF [8] and CrossDocked 2020 [35], are unsuitable for algorithm selection. These benchmarks provide either single-model submissions, aggregated scoring-function results, or affinity labels, but do not release per-algorithm pose predictions across a solver portfolio. Since AS requires instance-level performance profiles for multiple algorithms to define SBS, VBS, and supervised training labels, such datasets cannot be used without recomputing all predictions under a controlled protocol. For this reason, our evaluation focuses on *PoseX* and *PoseBusters*.

#### Preprocessing

Protein inputs are represented at the residue level. For each protein, we encode per-residue embeddings using ESM C 600 M [24], yielding a sequence of vectors $\textbf{x}_P \in \mathbb {R}^{|\text {residues}| \times 1152}$. Ligand inputs are encoded using ChemBERTa-77 M-MLM [25] to a single pooled embedding $\textbf{x}_L \in \mathbb {R}^{384}$. Both models supply pretrained molecular language representations. ESM C provides residue-level vectors that capture evolutionary and structural regularities, offering a high signal-to-noise representation without requiring supervised pocket annotations. ChemBERTa supplies a chemically informed ligand embedding that captures functional groups and local reactivity patterns more reliably than handcrafted descriptors. Using these pretrained models allows MolAS to operate in the small-data regime by leveraging representations already optimised on large biomolecular corpora, reducing the need for task-specific feature engineering. For each complex, the final input therefore consists of a pair of a protein residue embedding $\textbf{x}_P$ and a ligand embedding $\textbf{x}_L$.

### Model architecture

The encoder *f* consists of an attentional pooler applied to the protein embeddings, followed by a ligand-protein fusion module. The attention layer compresses the residue-level representations into a single protein vector, which is then concatenated with the ligand embedding and linearly projected into a lower-dimensional joint space.

Inspired by the demonstrated stability and variation preservation of ResNet [36], the fused representation is passed to a residual MLP that outputs per-algorithm performance scores. The decoder *g* comprises three consecutive residual blocks, each containing a linear layer, batch normalisation, and ReLU activation with a skip connection. The outputs of all residual blocks are concatenated and processed by a final linear projection head to produce the predicted score vector $\hat{\textbf{s}}\in \mathbb {R}^m$ over the algorithm portfolio.

### Scoring function

#### Geometric accuracy

**Fig. 2:**
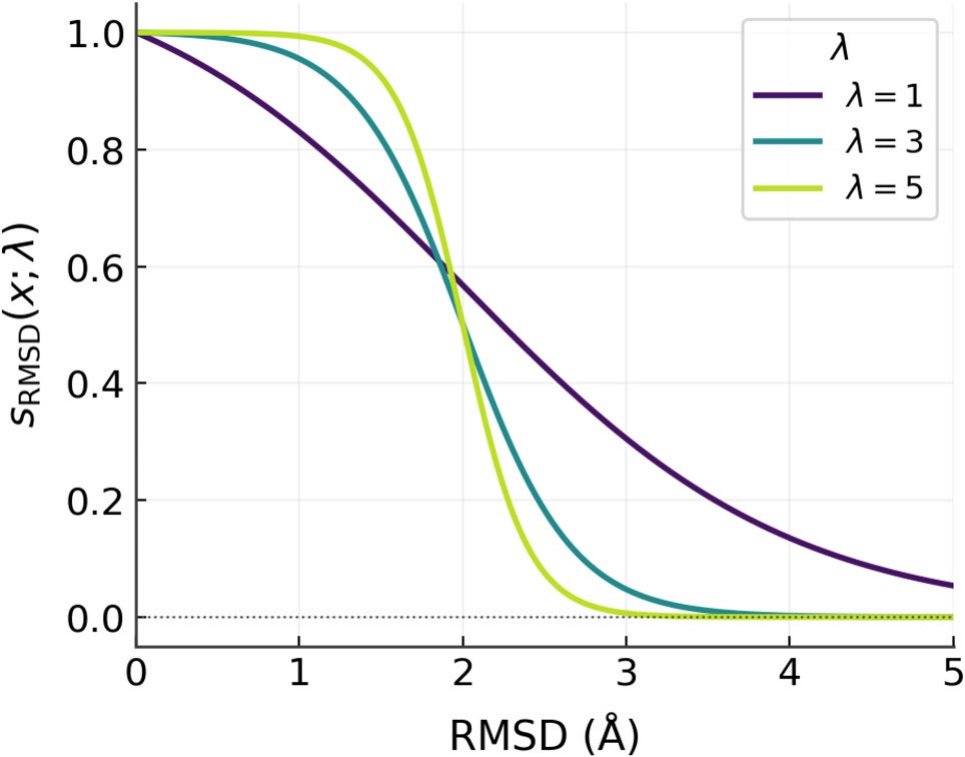
$$s_{RMSD}(x;\lambda )$$ curves when $$\lambda \in \{1, 3, 5\}$$

RMSD measures the spatial distance from a predicted pose to the crystal reference in Angstrom (Å). A pose with RMSD $\le 2$ Å is typically considered geometrically valid. The exponential scoring function used by MC-GNNAS-Dock [21] captures this diminishing returns in RMSD improvements but assigns zero value to all poses with RMSD above a hard threshold ($M = \ln 11 \approx 2.39$), thereby neglecting informative differences between moderately poor poses. To address this, we propose a smooth sigmoid-based score function:

4 $$\begin{aligned} s_{\textrm{RMSD}}(x;\lambda ) \;=\; \frac{1 + e^{-2\lambda }}{1 + e^{\lambda (x - 2)}}, \qquad \lambda> 0, \end{aligned}$$

which is centred at the critical threshold of 2 Å, where the maximum gradient occurs, ensuring the model rewards borderline improvements most strongly, while gains or losses away from this boundary yield diminishing marginal effect (Fig. 2). The parameter $\lambda$ controls the sensitivity of the score to changes in RMSD, and is set to $\lambda =3$ with an ablation conducted in "Ablation" section.

#### Physicochemical plausibility

The PB-validity score (PoseBusters-validity) of a pose is evaluated by a binary accept-reject gate that mirrors current practice in crystallographic validation:

5 $$\begin{aligned} s_{\text {PB}} \ =\ {\left\{ \begin{array}{ll} 1 \quad & \text { passes all } 18 \text { PoseBusters checks,} \\ 0 & \text {otherwise,} \end{array}\right. } \end{aligned}$$

which ensures that no geometrically plausible yet chemically impossible pose can receive a decent score. Our final score is then the RMSD score gated by PB-validity

6 $$\begin{aligned} s = s_{\text {RMSD}} \cdot s_{PB}. \end{aligned}$$

**Algorithm 1:**
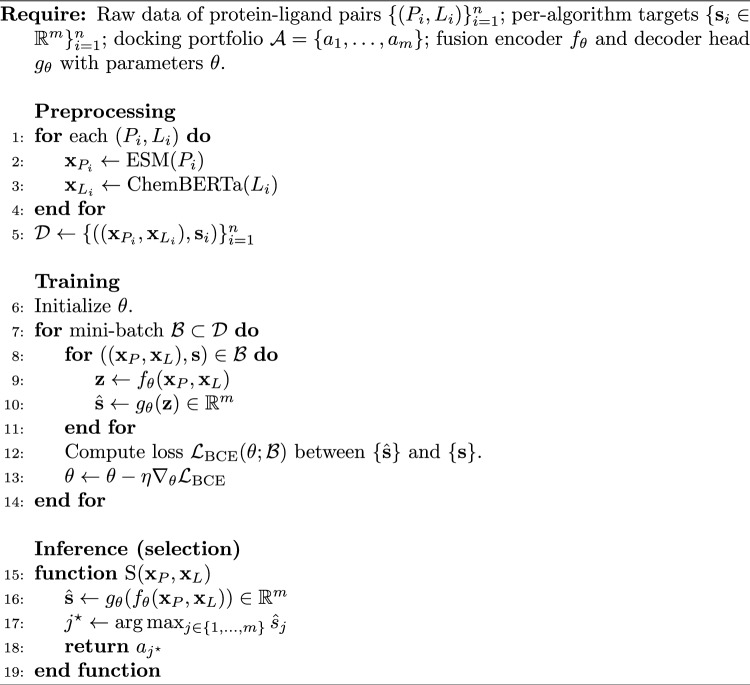
MolAS

### Training objective

MolAS is trained to predict per-algorithm gated score $s_a = s_{\textrm{RMSD},a}\cdot s_{\textrm{PB},a}$. Since multiple algorithms may simultaneously achieve high scores on the same complex, outputs are modelled independently per algorithm using sigmoid activations rather than a softmax normalisation across algorithms.

The loss uses binary cross-entropy with logits as a calibration objective for these bounded, multi-label targets:

$$\mathcal {L}_{\text {BCE}}(\hat{\textbf{s}}, \textbf{s}) = - \sum _{a \in \mathcal {A}} \Big [\, s_a \log \sigma (\hat{s}_a) + (1 - s_a)\log \bigl (1 - \sigma (\hat{s}_a)\bigr ) \Big ],$$

where $\hat{s}_a$ is the logit output and $\sigma$ is the logistic sigmoid. BCE-with-logits is a proper scoring rule for Bernoulli targets and naturally supports soft labels $s_a\in [0,1]$, yielding stable optimisation for bounded success-type scores. Multi-class cross-entropy is not appropriate because it would enforce competition between algorithms via normalisation, even when several solvers achieve similarly high scores on the same instance. Mean-squared error regression was also tested as a direct regression objective for $\textbf{s}$ but did not yield systematic improvements in this setting; ranking-aware alternatives (pairwise logistic and listwise NDCG@3) were explored as auxiliary objectives (Table 6) and likewise did not provide consistent gains. Training minimises the mean loss over mini-batches.

The procedures of MolAS are summarised in Alg. 1.

### Experimental setup

#### Implementation details

All models are implemented in Python using PyTorch. Docking outputs for PoseX and Astex are obtained from the official PoseX benchmark release [32], while those for PoseBusters are taken from the official PoseBusters release [33]. The code and pretrained weights of the ESM-C 600 M model are sourced from [37]. Models are trained using the Adam optimiser with a cosine learning-rate schedule and early stopping based on validation accuracy. All experiments are conducted on a dual-socket server equipped with two AMD EPYC 7763 CPUs and eight NVIDIA RTX 3090 GPUs, running Ubuntu 22.04.5 LTS.

#### Reporting protocol

We evaluate MolAS under two standard AS baselines. The single best solver (SBS) is the single docking method achieving the highest mean performance on the tested benchmark. The virtual best solver (VBS) is an oracle that chooses the highest-scoring method for each complex, i.e., the theoretical maximum or limit. In addition to absolute accuracy, we report the fraction of the VBS-SBS gap closed, denoted as $\%\frac{\text {AS}-\text {SBS}}{\text {VBS}-\text {SBS}}$, which quantifies the actionable improvement achieved by MolAS relative to the upper bound imposed by the portfolio.

For interpretability, some results involve Selected@K and VBS@K, which summarise the behaviour of the selector beyond the top choice. Here, Selected@K denotes the *algorithm* ranked $K^{th}$ by MolAS selection frequency over the tested benchmark, e.g., the second most frequently selected algorithm when $K=2$. VBS@K denotes a virtual *selector* that selects the $K^{th}$ best algorithm for each instance.

Two evaluation regimes are used. For in-domain learning, each benchmark is evaluated using a 5-fold split with an 8-2 train-test ratio per fold; results report the mean over all folds. For cross-benchmark learning, the entire source benchmark is used for training and the entire target benchmark for testing, with no overlap between complexes. All metrics are reported for success rates of both strict (RMSD $\le 1$ Å & PB-valid) and relaxed (RMSD $\le 2$ Å & PB-valid) success criteria.

## Results

We report MolAS’ in-domain performance ("In-domain learning" section), analyse its failure cases and their underlying causes ("Operational boundaries of MolAS" section), and evaluate cross-benchmark generalisation ("Cross-benchmark generalisation" section). We further compare MolAS with the prior GNN-based MC-GNNAS-Dock framework ("Comparison to MC-GNNAS-Dock" section) and provide architectural and data-driven ablations ("Ablation" section).

### Benchmark performance

**Table 3:** Averaged 5-fold MolAS performance v.s. SBS across benchmarks

Dataset	Post-process	SBS	$(\%) \text {RMSD}\le 1\,{{{\AA }}}\ \& \ \text {PB-valid}$			$(\%) \text {RMSD}\le 2\,{{{\AA }}}\ \& \ \text {PB-valid}$		
			SBS	MolAS	$\%\frac{AS-SBS}{VBS-SBS}$	SBS	MolAS	$\%\frac{AS-SBS}{VBS-SBS}$
With-and-without relaxation trained together								
MOAD-curated	Mixed	Uni-Mol	43.41	**50**.01*	**17**.**42**	68.17	**74**.68*	**22**.**40**
PoseX + Astex	Mixed	SurfDock (relax)	50.24	**58**.61*	**21**.**42**	74.08	**79**.8*	**24**.**24**
PoseX-SD	Mixed	SurfDock (relax)	50.08	**51**.**62**	**3**.**66**	74.21	**74**.**63**	**1**.**67**
PoseX-CD	Mixed	SurfDock (relax)	49.08	**66**.39*	**45**.**50**	73.02	**87**.50*	**61**.**28**
PoseBusters	Mixed	AutoDock	34.34	**36**.**69**	**8**.**90**	51.17	**54**.**91**	**11**.**87**
With-and-without relaxation trained separately								
PoseX + Astex	No	Boltz1x	42.48	**57**.05*	**32**.**12**	61.02	**76**.58*	**42**.**67**
	Relaxation	SurfDock (relax)	50.24	**58**.94*	**23**.**32**	74.08	**80**.84*	**28**.**99**
PoseX-SD	No	Uni-Mol	40.73	**43**.**51**	**5**.**56**	60.53	**62**.**62**	**5**.**41**
	Relaxation	SurfDock (relax)	50.08	**50**.**22**	**0**.**37**	74.21	73.93	−1.10
PoseX-CD	No	Boltz1x	44.82	**61**.66*	**41**.**48**	65.16	**83**.38*	**58**.**29**
	Relaxation	SurfDock (relax)	49.08	**66**.99*	**49**.**27**	73.02	**88**.41*	**66**.**43**
PoseBusters	No	AutoDock	34.34	**36**.**44**	**11**.**67**	51.17	**53**.**97**	**9**.**83**
	MM-min	Gold (MM-min)	27.58	26.87	−3.24	45.10	**45**.**81**	**2**.**34**

Columns list PoseBusters-validated pose rates within 1 Å and 2 Å RMSD, and the VBS-SBS gap closed. Bold marks improvement over SBS, and an improvement with * indicates $p<0.05$ (paired test between MolAS and SBS)

#### In-domain learning

Table 3 reports within-distribution performance across benchmarks. On the composite *MOAD-curated* set, MolAS attains success rates of 50.01% under the strict (RMSD $\le 1$ Å & PB-valid) criterion and 74.68% under the relaxed (RMSD $\le 2$ Å & PB-valid) criterion, improving over the SBS (Uni-Mol Docking V2) by 6.6% and 6.51% respectively. These gains close roughly 17–23% of the VBS–SBS gap. Improvements are more pronounced on *PoseX-CD*, where MolAS closes approximately 45% and 61% of the gap under the strict and relaxed criteria, consistent with cross-docking acting as a stronger receptor-mismatch stress test that induces larger shifts in solver rankings. On the hybrid *PoseX + Astex* dataset, absolute gains of 8.38% (strict) and 5.72% (relaxed) correspond to 21–24% gap closure, indicating robust behaviour under substantial structural and chemical diversity when trained on comparably heterogeneous data. All these improvements above SBS are statistically significant at $p < 0.05$ (paired test).

The improvements on *PoseBusters* from (34.3%, 51.2%) to (36.7%, 54.9%) are relatively marginal. A similar pattern is also seen on *PoseX-SD*, where MolAS performs closely to the SBS—(50.1%, 74.2%) v.s. (51.6%, 74.6%). In these regimes, the VBS–SBS gap is comparatively smaller and solver hierarchies are more stable, leaving less headroom for selection-driven gains; correspondingly, MolAS tends to default to the dominant solver more often, which is analysed in "Operational boundaries of MolAS" section.

To further quantify the effect of relaxation, we compared models trained on mixed versus post-process-separated datasets using the VBS–SBS gap closed as the metric of interest, as SBS performance varies between regimes. The post-process flag indicates similar trends. For the hybrid *PoseX + Astex* benchmark, separating the relaxation regimes yielded higher performance in both subsets, suggesting that independent modelling of (relax) and un(relax) outputs slightly improves consistency. For *PoseX-SD*, non-uniform trends are observed: the non-(relax) regime yields an incremental improvement relative to the mixed baseline, while the (relax) regime is marginally worse; given the small magnitude, these are rather considered as non-systematic fluctuations. *PoseX-CD* maintains strong performance across all conditions, again with only minor fluctuations that are not systematic across folds. In contrast, on *PoseBusters*, MolAS performs comparably to mixed data in the non-(MM-min) subset but shows a marked drop under the (MM-min) regime, likely reflecting the different energy-minimization procedure used in that dataset.

Overall, MolAS outperforms the SBS over most tested scenarios and remains robust across post-processing regimes, while exhibiting clear protocol-specific failure patterns (e.g., on *PoseX-SD*), which are investigated subsequently.

**Fig. 3:**
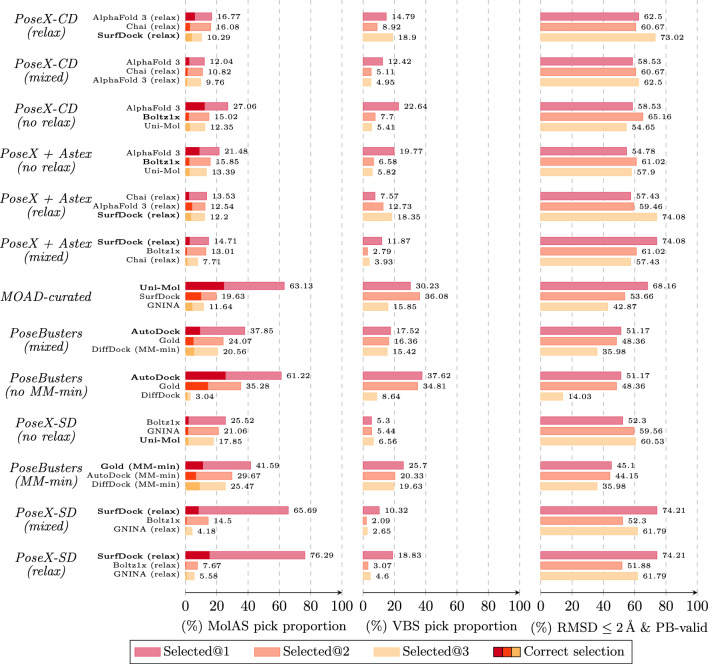
Selection distribution diagnostics across benchmarks. For each benchmark (rows), the left panel reports MolAS selection frequencies for its top-3 most frequently chosen solvers; the middle panel reports the VBS frequencies for the same solvers; and the right panel reports their realised success rates under the $\textrm{RMSD}\le 2~{{{\AA }}}\ \& \ \textrm{PB}$-valid criterion. Darker segments mark cases where MolAS’ top-1 choice agrees with the VBS, and the SBS for each benchmark is shown in bold. The figure highlights two regimes: (i) *selection collapse*, where MolAS concentrates on a single solver substantially more than the VBS and gains over SBS are negligible, and (ii) *distributed selection*, where MolAS allocates mass across multiple competitive solvers and closes a larger fraction of the VBS–SBS gap even when exact oracle matching is below 50%

**Fig. 4:**
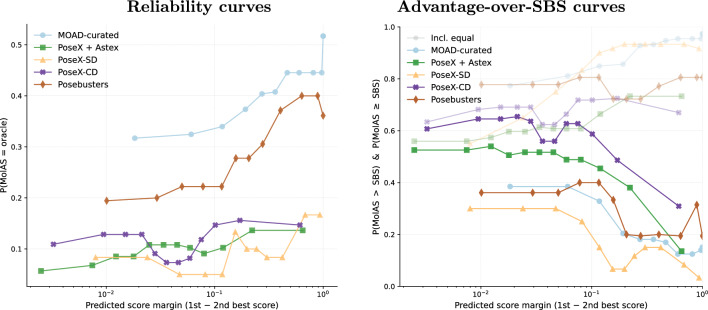
Confidence diagnostics via MolAS score margins (12 equal-mass bins). Left: reliability, $\mathbb {P}(\text {MolAS}=\text {VBS})$, as a function of the predicted margin between the top-1 and top-2 solvers. Right: advantageness over SBS, $\mathbb {P}(s_{\text {MolAS}}> s_{\text {SBS}})$ (solid) and $\mathbb {P}(s_{\text {MolAS}} \ge s_{\text {SBS}})$ (faded), over the same bins. Across benchmarks, larger margins tend to increase oracle agreement but often coincide with re-selection of the SBS, so margin is a benchmark-dependent confidence proxy rather than a universally calibrated indicator of improvement over SBS

#### Operational boundaries of MolAS

The behaviour of MolAS deteriorates on *PoseX-SD* and *PoseBusters* under the post-processing regimes and in several cross-benchmark settings. We therefore examined these regimes in more detail, (i) focusing on how the selector distributes its choices across the portfolio, (ii) whether those choices are conditionally reliable, and (iii) how the embedding space and algorithm landscape affect the performance.

Figure 3 characterises a key failure mode of MolAS: *selection collapse*. In difficult regimes such as *PoseX-SD (relax)* and *PoseBusters (MM-min)*, the selector concentrates most probability mass on a single solver far more aggressively than the VBS does, effectively behaving like a near-constant policy. This collapse is consistent with regimes where (i) the SBS dominates the oracle landscape or (ii) multiple solvers are near-tied, so that the embedding-derived signal is insufficient to support stable instance-wise differentiation; in both cases, little headroom remains for selection gains beyond SBS.

In contrast, when MolAS closes a substantial fraction of the VBS–SBS gap, its selections remain distributed across several competitive solvers and better reflect the oracle’s multi-modality. Notably, exact oracle matching can remain below 50% while still yielding sizable gains, because the relevant success region is often *flat near the top*: selecting a near-best solver is frequently sufficient when multiple solvers achieve similar composite success. This is consistent with the observation that VBS@2 and VBS@3 already exceed SBS performance on these benchmarks (Appendix A.2).

Figure 4 then links these outcomes to the predicted score margin between the top two solvers. Reliability $\mathbb {P}(\text {MolAS}=\text {VBS})$ generally increases with margin, but the probability of strictly outperforming SBS often decreases at large margins. This indicates that high margins frequently correspond to confident re-selection of the SBS (or another dominant solver) rather than confident identification of a better alternative. Consequently, margin behaves as a partially informative, benchmark-dependent confidence signal: it can reflect oracle agreement on low-entropy regimes, but it is not a reliable monotone indicator of improvement over SBS under high-entropy or near-tie regimes.

We additionally examined whether MolAS failures correlate with gross changes in the embedding space. The PCA spectra, t-SNE projections, centroid-distance heatmaps, and quantitative cluster/oracle statistics are reported in Appendix A.2.2 (Fig. 13, Fig. 14, Table 8). Briefly, the PCA spectra are broadly similar across benchmarks, indicating no obvious low-rank collapse. However, embedding-based separability remains weak overall, and in the most difficult regimes (e.g., PoseX-SD), high oracle diversity coincides with poor separability, consistent with the observed tendency of MolAS to collapse towards an SBS-like strategy.

In summary, MolAS is most effective when multiple algorithms are competitive and occupy at least partially distinct embedding regions. When embeddings fail to separate algorithms, the selector becomes overconfident and defaults to a near-SBS policy. This defines a clear operational boundary for learned docking algorithm selection.

**Table 4:** Cross-benchmark results of MolAS performance v.s. SBS (of the test set) across benchmark pairs

Train	Test	Post-process	SBS	$\%\le 1\,{{{\AA }}}\ \& \ \text {PB-valid}$			$\%\le 2\,{{{\AA }}}\ \& \ \text {PB-valid}$		
				SBS	MolAS	$\%\frac{AS-SBS}{VBS-SBS}$	SBS	MolAS	$\%\frac{AS-SBS}{VBS-SBS}$
With-and-without relaxation trained together									
PoseX-SD	PoseX-CD	Mixed	SurfDock (relax)	49.09	**50**.**53**	**3**.**79**	73.02	**74**.**01**	**4**.**19**
PoseX-CD	PoseX-SD	Mixed	SurfDock (relax)	50.07	44.63	−12.96	74.2	62.2	−47.81
PoseX-SD	Astex	Mixed	SurfDock (relax)	69.41	65.88	−11.54	89.41	87.06	−22.19
PoseX-CD	Astex	Mixed	SurfDock (relax)	69.41	65.88	−11.54	89.41	82.53	−66.67
With-and-without relaxation trained separately									
PoseX-SD	PoseX-CD	No	Boltz1x	44.82	40.7	−10.14	65.17	59.98	−16.61
		Relaxation	SurfDock (relax)	49.09	**50**.**0**	**2**.**50**	73.02	72.94	−0.35
PoseX-CD	PoseX-SD	No	Uni-Mol	40.73	38.91	−3.64	60.53	57.04	−9.03
		Relaxation	SurfDock (relax)	50.07	42.68	−18.53	74.2	59.55	−58.37
PoseX-SD	Astex	No	Uni-Mol	77.65	58.82	−84.25	85.88	75.29	−75.00
		Relaxation	SurfDock (relax)	69.41	67.06	−7.68	69.41	87.06	−22.19
PoseX-CD	Astex	No	Uni-Mol	77.65	56.47	−94.77	85.88	70.59	−108.29
		Relaxation	SurfDock (relax)	69.41	67.06	−7.68	69.41	81.18	−77.71

Columns list PoseBusters-validated pose rates within 1 Å and 2 Å RMSD, and the VBS-SBS gap closed. Bold marks improvement over SBS, and an improvement with $*$ indicates $p<0.05$ (paired test between MolAS and SBS)

**Table 5:** Protocol-pair solver-ranking stability

Post-Process	Benchmark pair	*m*	Spearman $\rho$	Kendall $\tau _b$	$J_1$	$\overline{J}$
Mixed	(PoseX-SD, PoseX-CD)	48	0.841	0.679	1.000	0.660
	(PoseX-SD, Astex)	48	0.858	0.702	1.000	0.715
	(PoseX-CD, Astex)	48	0.880	0.729	1.000	0.716
No	(PoseX-SD, PoseX-CD)	24	0.843	0.710	0.000	0.647
	(PoseX-SD, Astex)	24	0.804	0.652	1.000	0.720
	(PoseX-CD, Astex)	24	0.849	0.681	0.000	0.686
Relaxation	(PoseX-SD, PoseX-CD)	24	0.817	0.645	1.000	0.669
	(PoseX-SD, Astex)	24	0.857	0.710	1.000	0.731
	(PoseX-CD, Astex)	24	0.856	0.717	1.000	0.715

For each protocol pair, Spearman’s $\rho$ and Kendall’s $\tau _b$ are computed between solver rankings induced by mean per-solver scores. $J_1$ is the top-1 Jaccard overlap, and $\overline{J}=\frac{1}{m}\sum _{k=1}^{m} J_k$ is the mean top-*k* Jaccard overlap over $k\in \{1,\dots ,m\}$

**Fig. 5:**
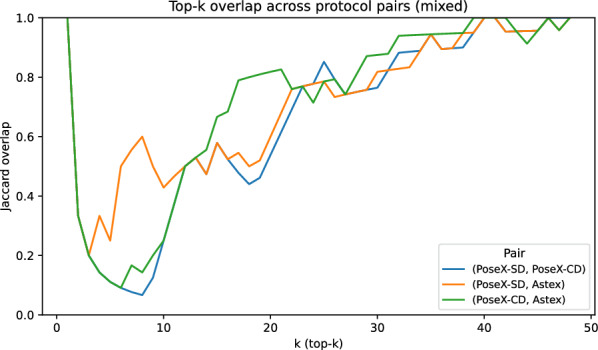
Top-*k* overlap of solver rankings across docking protocols. Each curve reports the Jaccard similarity between the sets of the top-*k* solvers under a benchmark pair (*p*, *q*), over $k=1,\dots ,m$, where $m=|\mathcal {A}|$ is the number of solvers. Higher overlap indicates a more stable solver hierarchy across protocols, while low overlap at small *k* highlights instability among the highest-ranked solvers

#### Cross-benchmark generalisation

The cross-benchmark results in Table 4 indicate that MolAS does not achieve meaningful generalisation across docking distributions. Although a few *PoseX SD* $\rightarrow$*CD* transfers produce small positive gap closures (3.79% and 4.19% in the mixed regime, 2.50% in the relax-only regime), none of these improvements reach statistical significance, and all are numerically marginal. Without statistical support, these fluctuations are better interpreted as noise rather than evidence of generalisable behaviour.

In contrast, the majority of cross-distribution transfers show consistent and large degradations, including around $13\%$–$48\%$ *VBS–SBS gap widening* for *PoseX CD*$\rightarrow$*SD*, $7\%$–$84\%$ for *PoseX-SD*$\rightarrow$*Astex*, and up to $108\%$ gap widening for *PoseX-CD*$\rightarrow$*Astex*. These failures span both the 1 Å and 2 Å thresholds and persist regardless of whether relaxation and non-relaxation data are trained jointly or separately. The overall pattern is that when the target benchmark differs materially in curation and protocol, including the distribution of targets/ligands and the evaluation pipeline, transfer degrades towards the SBS baseline or worse. Consistently, in poorly transferring regimes, the selector’s outputs contract onto a small subset of methods (often a single dominant solver), diverging from the oracle distributions of the target benchmarks.

To quantify the protocol dependence underlying these observations, Table 5 reports explicit solver-hierarchy stability diagnostics across benchmark pairs, separately for the mixed, no-post-processing, and relaxation settings. Across protocol pairs, rank correlations of the solver ordering induced by mean scores are moderate-to-high ($\rho \in [0.80,0.88]$, $\tau _b\in [0.64,0.73]$), indicating that the global ranking is not arbitrarily permuted. However, these global correlations do not imply stability of the upper tail that determines oracle labels for algorithm selection. The top-*k* overlap diagnostics reveal materially weaker agreement among the highest-ranked solvers: the mean top-*k* overlap $\overline{J}$ ranges from 0.65 to 0.73, and the top-1 overlap $J_1$ is zero for some no-post-processing benchmark pairs, implying that even the identity of the best solver can change across protocols.

The top-*k* overlap curves (Fig. 5) provide a complementary view of where ranking disagreement concentrates. Across benchmark pairs, overlap is typically high at $k=1$ (shared best solver in the mixed setting; Table 5) but drops substantially for small-to-moderate *k* (e.g., top-5/top-10), before increasing steadily as *k* grows. This indicates that protocols agree more on the single best method than on the set of near-best contenders, i.e., protocol shifts primarily reorder the upper tail rather than arbitrarily permuting the full portfolio. Such near-top reordering is particularly relevant for algorithm selection, because oracle labels are mostly determined by differences among a small set of high-performing solvers; perturbations within this set can therefore reduce transferability even when global rank correlations remain high. The no-post-processing and relaxation settings exhibit the same qualitative behaviour; full top-*k* curves for these settings are reported in Appendix A.2.3.

Taken together, Tables 4 and 5 support a conservative interpretation of the transfer failures: MolAS does not learn an invariant mapping from molecular geometry to the optimal solver under a fixed portfolio; instead, it fits decision boundaries aligned to the training protocol’s score landscape and solver hierarchy. When the target protocol induces a different upper-tail hierarchy (even if the global ordering remains broadly correlated), the learned decision surface becomes misaligned with the target oracle, yielding systematic negative transfer. This limitation is intrinsic to portfolio-based docking AS: supervision is defined by protocol-specific performance profiles, and cross-protocol robustness cannot be assumed without additional mechanisms (e.g., explicit domain adaptation, calibration on the target protocol, or selectors designed to be stable under hierarchy perturbations).

Finally, it is useful to distinguish cross-protocol instability from within-protocol oracle diversity. The VBS entropy in Table 8 summarises the within-protocol dispersion of oracle winners and indicates that, within each benchmark, multiple solvers can be competitive. The protocol-pair stability diagnostics above instead quantify how the induced hierarchy shifts across protocols; both aspects are relevant, but they capture different failure modes for transfer.

**Fig. 6:**
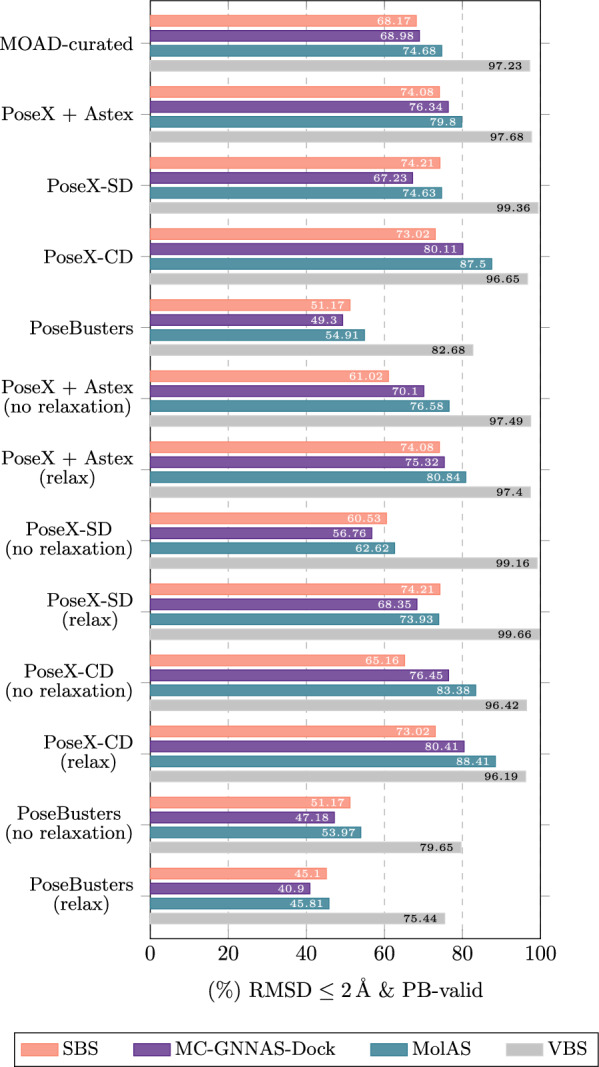
Comparison among SBS, MC-GNNAS-Dock, MolAS, and VBS in averaged 5-fold results in in PoseBusters-validated pose rate within 2 Å RMSD across benchmarks

### Comparison to MC-GNNAS-Dock

We benchmarked MC-GNNAS-Dock [21] across the same distributions to assess whether MolAS offers a substantive improvement over a heavier, GNN-based selector. As summarised in Fig. 6, MolAS consistently outperforms MC-GNNAS-Dock across all evaluated benchmarks. The gains range from approximately $3.5-8\%$ in the $2~{{{\AA }}}$ & PB-validity metric. Notably, these improvements are achieved with a substantially smaller model: $\sim 638\text {k}$ parameters for MolAS versus $\sim 3.2\text {M}$ for MC-GNNAS-Dock. This suggests that in the current data regime, lightweight embedding-level architectures exploit available signal more efficiently than deeper graph-based models, aligning with the earlier ablation results that point to a data-limited rather than architecture-limited setting.

**Table 6:** Component-wise ablation of architectural modules and optimisation objectives based on the MolAS baseline, with the default choices indicated in the brackets

Ablated items	$\%\le$x Å & PB-valid			
	MOAD-curated		PoseX + Astex	
	$x=1$	$x=2$	$x=1$	$x=2$
SBS	43.41	68.17	50.24	74.08
MolAS	50.01	74.68	58.61	79.80
GNN encoder (MolAS: None)				
GCN-GAT-GINE	48.38$^{\pmb {\downarrow }}$	71.34$^{\pmb {\downarrow }}$	56.81$^{\pmb {\downarrow }}$	78.81$^{\pmb {\downarrow }}$
EGNN-GAT-GINE	47.37$^{\pmb {\downarrow }}$	71.81$^{\pmb {\downarrow }}$	57.19$^{\pmb {\downarrow }}$	78.67$^{\pmb {\downarrow }}$
Protein attention heads (MolAS: 1)				
2	48.22$^{\pmb {\downarrow }}$	72.70$^{\pmb {\downarrow }}$	58.37$^{\pmb {\downarrow }}$	79.90$^{\pmb {\uparrow }}$
4	49.01$^{\pmb {\downarrow }}$	72.92$^{\pmb {\downarrow }}$	58.70$^{\pmb {\uparrow }}$	79.85$^{\pmb {\uparrow }}$
Decoder (MolAS: 4 ResBlock(128, 256))				
6 ResBlock(128, 256)	48.19$^{\pmb {\downarrow }}$	71.75$^{\pmb {\downarrow }}$	59.93$^{\pmb {\uparrow }}$	80.27$^{\pmb {\uparrow }}$
2 ResBlock(128, 256)	47.81$^{\pmb {\downarrow }}$	71.56$^{\pmb {\downarrow }}$	59.13$^{\pmb {\uparrow }}$	80.37$^{\pmb {\uparrow }}$
4 ResBlock(256, 512)	48.79$^{\pmb {\downarrow }}$	72.29$^{\pmb {\downarrow }}$	58.23$^{\pmb {\downarrow }}$	79.28$^{\pmb {\downarrow }}$
4 ResBlock(64, 128)	48.57$^{\pmb {\downarrow }}$	71.94$^{\pmb {\downarrow }}$	57.71$^{\pmb {\downarrow }}$	80.42$^{\pmb {\uparrow }}$
Single linear layer	48.88$^{\pmb {\downarrow }}$	72.57$^{\pmb {\downarrow }}$	59.84$^{\pmb {\uparrow }}$	80.98$^{\pmb {\uparrow }}$
Scoring function (MolAS: $\lambda =3$)				
$\lambda = 1$	48.75$^{\pmb {\downarrow }}$	70.75$^{\pmb {\downarrow }}$	60.12$^{\pmb {\uparrow }}$	78.01$^{\pmb {\downarrow }}$
$\lambda = 5$	48.48$^{\pmb {\downarrow }}$	72.85$^{\pmb {\downarrow }}$	57.52$^{\pmb {\downarrow }}$	80.80$^{\pmb {\uparrow }}$
Ranking-aware loss (MolAS: BCE)				
BCE + PL	48.50$^{\pmb {\downarrow }}$	71.47$^{\pmb {\downarrow }}$	59.65$^{\pmb {\uparrow }}$	77.15$^{\pmb {\downarrow }}$
BCE + NDCG@3	48.47$^{\pmb {\downarrow }}$	72.32$^{\pmb {\downarrow }}$	59.70$^{\pmb {\uparrow }}$	80.51$^{\pmb {\uparrow }}$
BCE + PL + NDCG@3	48.22$^{\pmb {\downarrow }}$	71.25$^{\pmb {\downarrow }}$	60.12$^{\pmb {\uparrow }}$	78.01$^{\pmb {\downarrow }}$
Modality dropout (MolAS: both embeddings)				
Protein-only (embeddings)	45.58$^{\pmb {\downarrow }}$	68.83$^{\pmb {\downarrow }}$	53.83$^{\pmb {\downarrow }}$	74.93$^{\pmb {\downarrow }}$
Ligand-only (embeddings)	48.28$^{\pmb {\downarrow }}$	73.10$^{\pmb {\downarrow }}$	58.66$^{\pmb {\uparrow }}$	80.61$^{\pmb {\uparrow }}$
Protein-only (graph+embeddings) with GCN-GAT-GINE	46.71$^{\pmb {\downarrow }}$	69.55$^{\pmb {\downarrow }}$	52.93$^{\pmb {\downarrow }}$	73.93$^{\pmb {\downarrow }}$
Ligand-only (graph+embeddings) with GCN-GAT-GINE	48.38$^{\pmb {\downarrow }}$	73.10$^{\pmb {\downarrow }}$	58.66$^{\pmb {\uparrow }}$	80.61$^{\pmb {\uparrow }}$

$^{\pmb {\uparrow }}$denotes improvements over MolAS and $^{\pmb {\downarrow }}$denotes declinations

### Ablation

MolAS uses separate protein and ligand embeddings and does not explicitly encode protein–ligand 3D contact graphs. As discussed in prior analyses of bias and shortcut learning in structure-based ML benchmarks [38, 39], this design choice motivates explicit controls for whether performance is driven by a single modality or by protocol-specific artefacts. Table 6 therefore includes modality-dropout variants (protein-only, ligand-only, and full input), reported under the same evaluation protocols as the main model.

Across the two major benchmark sets, *MOAD-curated* and *PoseX + Astex*, parallel GNN encoder stacks (GCN–GAT–GINE, inspired by graphLambda [40], and an equivariant EGNN–GAT–GINE variant) fail to surpass the embedding-only model, indicating that pretrained residue-level ESM C and ligand ChemBERTa representations are already sufficient to support in-domain selection in this data regime. Increasing the number of attention heads produces only sub-percentage fluctuations with no consistent pattern. Decoder capacity exhibits a similarly limited effect, with deeper or wider residual stacks yielding only marginal gains on one benchmark at the expense of performance on the other. A single linear decoder achieves peak performance on the *PoseX + Astex* set under the $2~{{{\AA }}}$ & PB-validity criterion; however, its performance on the *MOAD-curated* benchmark decreases by approximately $2\%$, suggesting limited benefit from architectural scaling at the current data scale.

The modality-dropout controls further clarify how signal is distributed across inputs. In the embedding-only setting, removing either the protein or ligand embedding leads to a moderate degradation relative to the full model, indicating that neither modality alone trivially explains the selection labels. In the graph+embedding setting, removing protein information causes a substantially larger drop than removing ligand information, while removing ligand information has a smaller effect. Taken together, these ablations do not support a degenerate single-modality shortcut explanation on the presented benchmarks, while leaving open the possibility that explicit protein–ligand contact features could improve robustness under stronger distribution shifts (Sec. 5).

Sensitivity to the scoring-function hyperparameter is modest: altering the RMSD score rewarding parameter  from $\lambda =3$ to $\lambda =1$ or 5 shifts scores by at most $\sim 2\%$, and the directions of change differ across benchmarks, implying that MolAS is relatively insensitive to reasonable weighting choices in the current regime. Ranking-aware objectives, including pairwise logistic (PL), Normalized Discounted Cumulative Gain (NDCG@3), and their combinations with BCE, likewise produce only small variations with no consistent gain (Table 6).

These negative results are informative when viewed through the oracle-landscape diagnostics. On several benchmarks the oracle is high-entropy and shallow near the top: multiple solvers frequently achieve similar composite success scores, and small changes in post-processing can reorder winners. In such regimes, the supervision signal exhibits (i) *near-ties* among top solvers, (ii) *non-smooth ranking transitions* where small score perturbations flip the top-1 label, and (iii) *label noise induced by protocol differences* when the same complex is evaluated under different workflows. Together, these properties make strict ranking supervision brittle: pairwise/listwise losses treat many near-ties as hard constraints, amplifying noise without adding stable information beyond a calibrated success tendency. Consistently, increasing model capacity (deeper decoders or GNN encoders) does not yield systematic gains, suggesting that the limiting factor is not expressiveness but the stability and separability of the workflow-defined labels.

Taken together, the ablations support an evidence-backed hypothesis that MolAS is *data- and label-regime limited* rather than *architecture-limited*: when oracle entropy is high and top-rank boundaries are non-smooth, additional capacity or more aggressive ranking losses provide limited benefit and can degrade transfer across benchmarks. The baseline configuration therefore represents an appropriate balance of expressiveness and stability for the scale and heterogeneity of the available data.

## Discussion

### When is MolAS useful in practice?

MolAS is best interpreted as a *workflow-adaptive selector* rather than a universal docking policy. Practical use is most straightforward when a project fixes, for a period, a target domain (protein family and ligand chemistry), a solver portfolio, and an evaluation/post-processing pipeline. Under such a fixed workflow, a modest in-domain calibration set can be labelled by running the portfolio on that subset, after which MolAS can be trained to reduce expected regret relative to a static solver, e.g. SBS, by exploiting systematic instance-dependent differences among solvers.

Empirically, the strongest gains appear in regimes where the induced selection problem is non-trivial: the VBS–SBS gap is appreciable and oracle winners are not concentrated in a single method. In these settings, performance tends to saturate with hundreds to a few thousand labelled complexes, consistent with the observed gap closure on benchmarks such as *MOAD-curated* and *PoseX-CD*. Conversely, when the oracle landscape is effectively dominated by a single solver or when multiple solvers are near-indistinguishable under the evaluation protocol, headroom for selection is limited and MolAS tends to converge to an SBS-like policy, which is locally reasonable but yields negligible improvement.

Workflow changes define the main operational boundary. Cross-benchmark experiments indicate that changes in docking settings, dataset curation, or post-processing can shift solver scores and reorder solver rankings, thereby changing the induced supervision signal. In practice, this implies that selectors trained without explicit workflow descriptors should not be assumed to transfer reliably across pipelines; when the portfolio or protocol changes, re-labelling and retraining on the new workflow may generally be required. When oracle diversity is low or the VBS–SBS gap is small, an SBS fallback remains an appropriate default.

### What does MolAS reveal about docking algorithm selection?

MolAS was designed to separate representational considerations from workflow-defined effects. Across the reported ablations, increasing architectural capacity (including graph-based encoders) and changing optimisation objectives (including ranking-aware losses) did not yield systematic gains. Within the evaluated regime, this pattern suggests that simply scaling model complexity is not the dominant lever for improving docking AS, at least when strong pretrained protein and ligand embeddings are available and supervision is defined by a workflow-specific oracle.

Instead, performance is strongly associated with properties of the *workflow-defined oracle landscape*. Several regimes exhibit high oracle diversity and shallow separation among top solvers, so that small perturbations of scores can flip the top-1 label. Combined with protocol-induced ranking shifts under post-processing or benchmark changes, this creates a supervision signal that is effectively non-smooth and partially noisy with respect to molecular inputs alone. This diagnostic picture provides an evidence-backed hypothesis for the observed ablation failures: ranking-aware losses can over-emphasise unstable near-ties, and additional capacity can overfit workflow-specific characteristics without improving robustness, yielding limited or inconsistent benefit beyond calibrated success prediction.

Importantly, the observed protocol dependence is not an impossibility result. Stronger generalisation across workflows may be achievable if protocol variables are modelled explicitly, e.g. by conditioning the selector on workflow descriptors, by learning representations that are invariant across a family of protocols, or by applying explicit domain adaptation when transferring between pipelines. Related difficulties with out-of-distribution generalisation have been reported in algorithm selection for other domains when solver rankings shift under distributional change, motivating adaptation mechanisms rather than assuming invariance [41].

A further limitation is that MolAS does not represent explicit protein–ligand contact geometry. While modality-dropout controls indicate that both protein and ligand embeddings contribute on the evaluated benchmarks, explicit cross-molecular interaction features may be required for stronger robustness under protocol and distribution shifts, particularly when oracle-defined solver regimes depend on fine-grained contact patterns not captured reliably by unimodal embeddings.

### Future directions

Several directions follow naturally from these findings. First, protocol-aware selection appears necessary for reliable transfer: incorporating workflow descriptors, modelling protocol families jointly, or using explicit adaptation across pipelines offers a principled route to improving cross-protocol performance. Second, more robust confidence estimation could mitigate winner-take-all collapses by enabling abstention or controlled fallback to SBS-like policies when the selector’s margins are not informative under high-entropy regimes. Third, incorporating explicit interaction geometry (e.g., contact features or cross-molecular graphs) remains a plausible path to improving robustness, provided that evaluation protocols and label definitions are controlled carefully enough to separate representational gains from workflow-induced effects. Together, these considerations delimit a realistic scope for docking AS: substantial gains are achievable under fixed workflows with non-trivial oracle diversity, while generalisation across workflows is a modelling problem rather than a property that can be assumed.

## Supplementary Information

Supplementary material 1.

## Figures and Tables

**Fig. 7:**
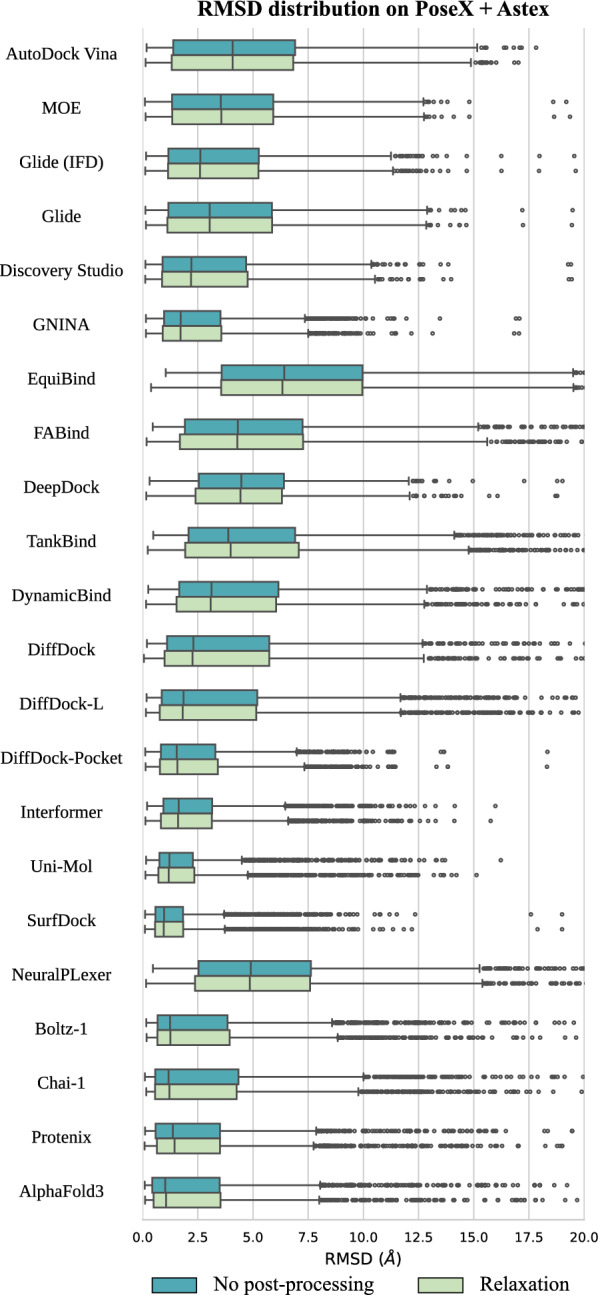
Distribution of ligand RMSD-to-reference for each method before (raw) and after PoseX relaxation, evaluated on PoseX + Astex. Boxes show the interquartile range with median; whiskers or outliers follow the standard boxplot convention

**Fig. 8:**
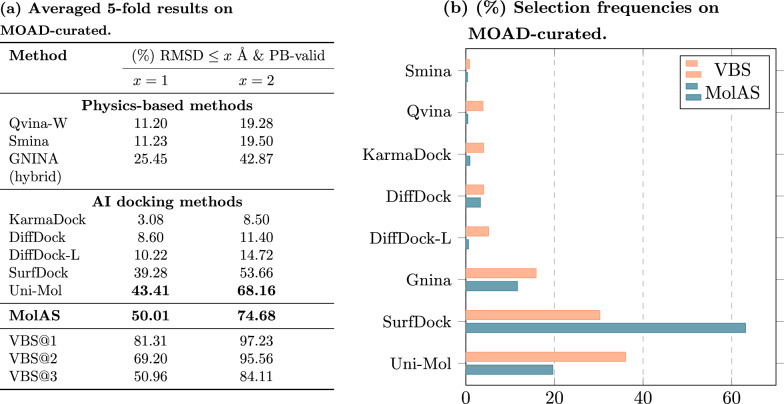
Table of performances over the algorithm portfolio (left) and histogram of MolAS/VBS selection frequencies (right) on MOAD-curated. In the table, the portfolio-wise best and MolAS performances are in bold. Performances are ordered by ascending percentage of poses below $2~{{{\AA }}}$ and PoseBusters-valid, while frequencies are ordered by ascending VBS frequency

**Fig. 9:**
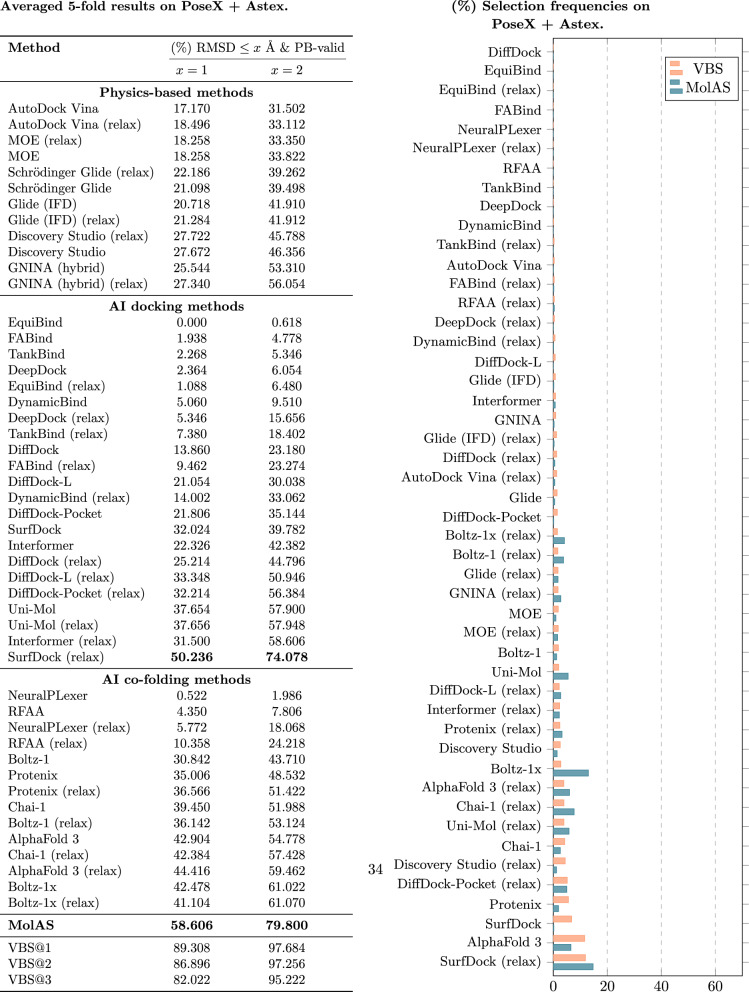
Table of performances over the algorithm portfolio (left) and histogram of MolAS/VBS selection frequencies (right) on PoseX + Astex. In the table, the portfolio-wise best and MolAS performances are in bold. Performances are ordered by ascending percentage of poses below $2~{{{\AA }}}$ and PoseBusters-valid, while frequencies are ordered by ascending VBS frequency

**Fig. 10:**
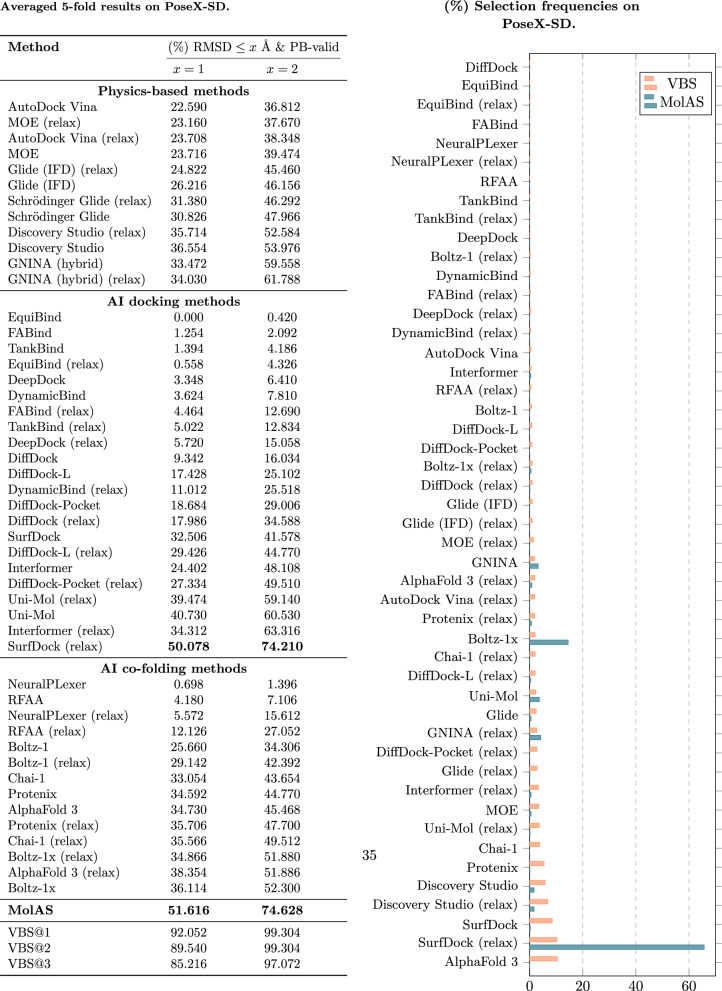
Table of performances over the algorithm portfolio (left) and histogram of MolAS/VBS selection frequencies (right) on PoseX-SD. In the table, the portfolio-wise best and MolAS performances are in bold. Performances are ordered by ascending percentage of poses below $2~{{{\AA }}}$ and PoseBusters-valid, while frequencies are ordered by ascending VBS frequency

**Fig. 11:**
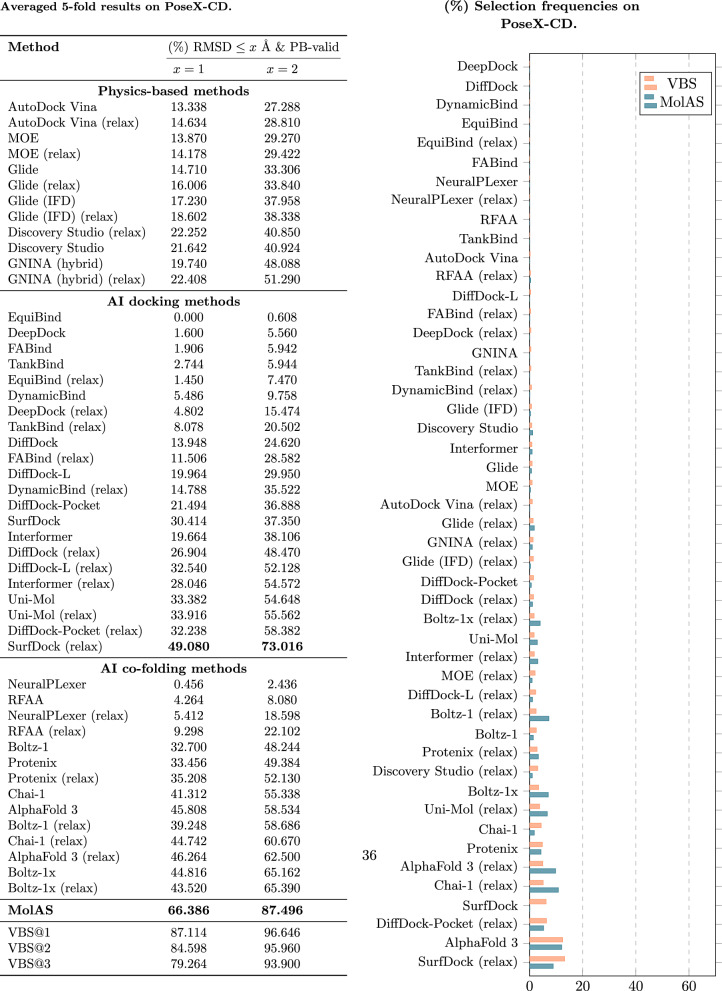
Table of performances over the algorithm portfolio (left) and histogram of MolAS/VBS selection frequencies (right) on PoseX-CD. In the table, the portfolio-wise best and MolAS performances are in bold. Performances are ordered by ascending percentage of poses below $2~{{{\AA }}}$ and PoseBusters-valid, while frequencies are ordered by ascending VBS frequency

**Fig. 12:**
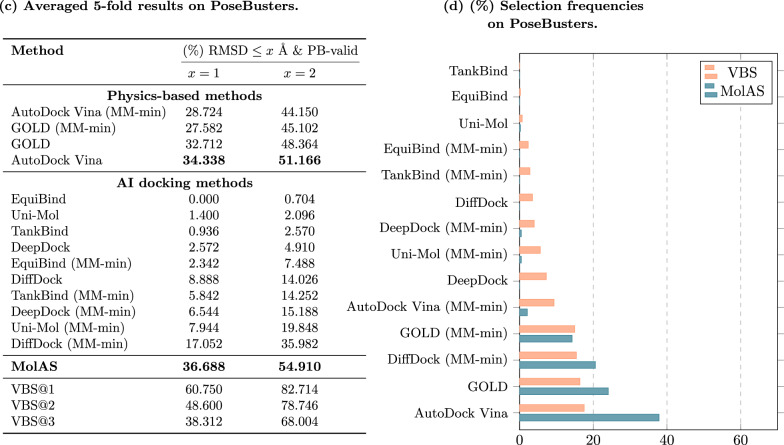
Table of performances over the algorithm portfolio (left) and histogram of MolAS/VBS selection frequencies (right) on PoseBusters. In the table, the portfolio-wise best and MolAS performances are in bold. Performances are ordered by ascending percentage of poses below $2~{{{\AA }}}$ and PoseBusters-valid, while frequencies are ordered by ascending VBS frequency

**Fig. 13:**
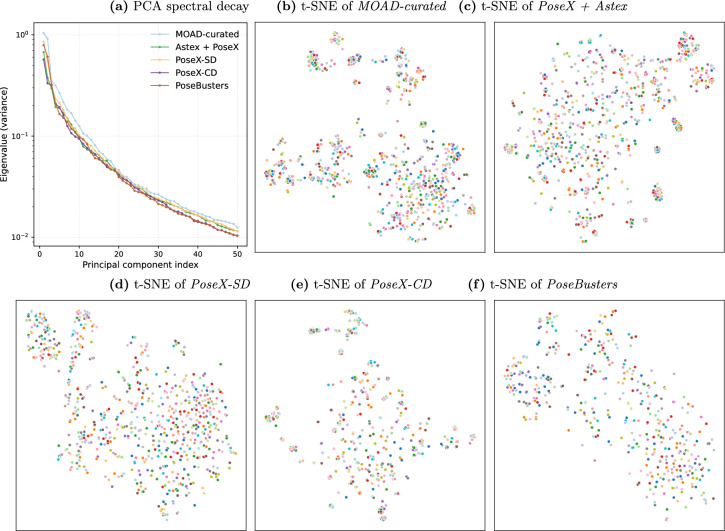
**a** Eigenvalue spectra via PCA. **b**–**f** t-SNE visualisations of embedding spaces across benchmarks. Each panel shows a 2D projection of embedding clusters coloured by their oracle-selected algorithm

**Fig. 14:**
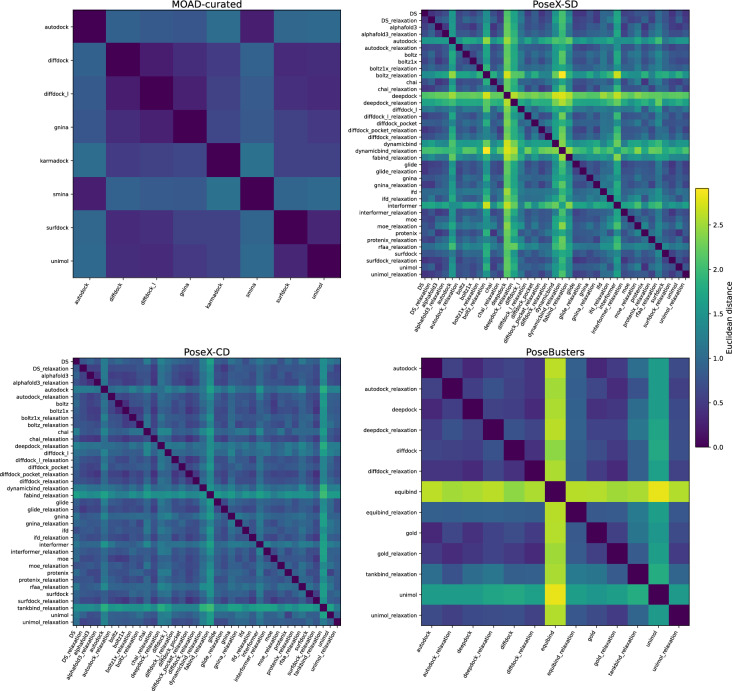
Pairwise Euclidean distances between embedding-space centroids of docking algorithms for each benchmark. Darker values indicate smaller distances; diagonal entries are zero by definition. Separate panels show MOAD-curated, PoseX-SD, PoseX-CD, and PoseBusters

**Fig. 15:**
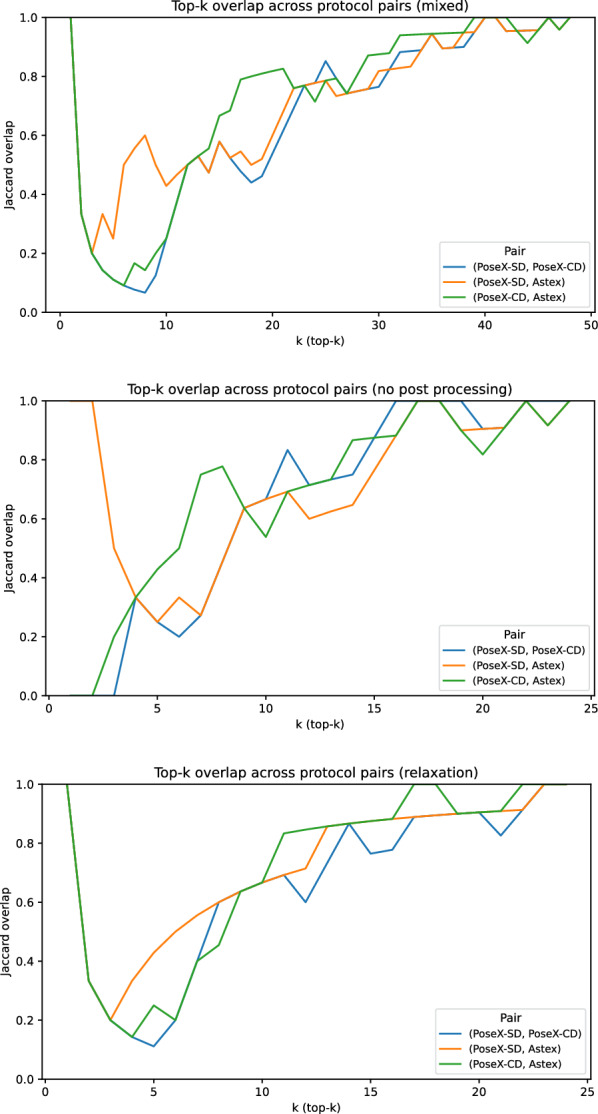
Top-*k* overlap of solver rankings across docking protocols under three post-processing settings (mixed, no post-processing, relaxation). Each curve reports the Jaccard similarity between the sets of the top-*k* solvers under a benchmark pair (*p*, *q*), for $k=1,\dots ,m$, where *m* is the number of solvers. Higher overlap indicates a more stable solver hierarchy across protocols, while low overlap at small *k* highlights instability among the highest-ranked solvers

## Data Availability

The dataset supporting the conclusions of this article including processed complexes, per-algorithm performances, and in-domain checkpoints is available on Zenodo at https://zenodo.org/records/17760688. The codes for generating embeddings, training and testing MolAS are available in the MolAS GitHub repository at https://github.com/BradWangW/MolAS.

## Appendix

### Supplements on dataset

**Effect of relaxation on RMSD and PoseBusters validity**

Figure 7 compares RMSD distributions before and after applying the PoseX relaxation step, and Table 7 summarises per-complex changes over $N{=}2115$ protein–ligand complexes (PoseX + Astex). Across all methods, relaxation rarely converts a PoseBusters-valid pose into an invalid one (0–36 complexes; $\le 1.7\%$), indicating that geometry correction is typically not detrimental to validity. In contrast, the number of validity improvements varies substantially by method, ranging from 5 (0.2%) to 1712 (80.9%) complexes, suggesting that some pipelines frequently produce strained or clash-prone raw geometries that are subsequently corrected. Across methods, larger mean $|\Delta \textrm{RMSD}|$ tends to coincide with more validity improvements (Pearson $r\approx 0.75$), consistent with the need for larger coordinate adjustments when resolving steric or geometric violations. Accordingly, relaxation can measurably shift RMSD-based outcomes for some methods, and results are therefore reported both without post-processing and with relaxation.

**Table 7:** Change in ligand RMSD-to-reference and PoseBusters validity after applying PoseX relaxation, evaluated on PoseX + Astex

Method	$|\Delta \textrm{RMSD}|$		PB-validity		
	Mean	IQR	$\mathrm {\#Improved}$	$\mathrm {\#Worsened}$	$\mathrm {\#Unchanged}$
AutoDock Vina	0.218	0.124	200	14	1901
MOE	0.075	0.068	6	30	2079
Glide (IFD)	0.073	0.070	5	28	2082
Schrödinger Glide	0.091	0.091	6	35	2074
Discovery Studio	0.075	0.078	16	27	2072
GNINA	0.108	0.119	152	36	1927
Equibind	1.185	0.820	1705	0	410
FABind	0.640	0.404	1223	1	891
DeepDock	0.299	0.314	1195	0	920
TankBind	0.685	0.396	1095	1	1019
DynamicBind	0.447	0.281	1712	1	402
DiffDock	0.329	0.192	1316	3	796
DiffDock-L	0.341	0.177	1142	10	963
DiffDock-Pocket	0.119	0.128	932	7	1176
Interformer	0.140	0.136	656	20	1439
Uni-Mol	0.123	0.120	97	25	1993
SurfDock	0.154	0.160	1069	8	1038
NeuralPLexer	0.655	0.421	1643	1	471
Boltz-1	0.215	0.134	440	18	1657
Chai-1	0.262	0.121	243	21	1851
Protenix	0.177	0.110	211	22	1882
AlphaFold3	0.210	0.111	230	18	1867

$\Delta \textrm{RMSD} = \textrm{RMSD}_{\text {relax}} - \textrm{RMSD}_{\text {raw}}$. “PB-validity” counts the number of complexes whose PoseBusters status improved (invalid $\rightarrow$ valid), worsened (valid $\rightarrow$ invalid), or remained unchanged

### Supplemental results

#### Detailed performance over portfolio

Detailed performances over algorithm portfolios and MolAS/VBS selection frequencies over *MOAD-curated*, *PoseX + Astex*, *PoseX-SD*, *PoseX-CD*, and *PoseBusters* are shown in Figs. 8, 9, 10, 11, and 12.

#### Embedding-space diagnostics (PCA/t-SNE/centroids)

We examined whether MolAS failures correlate with gross differences in the pooled protein+ligand embedding space using PCA spectra, t-SNE projections, and solver-wise centroid statistics (Figs. 13, 14, Table 8). Across benchmarks, the PCA eigenvalue spectra are broadly similar, with comparable decay and no evidence of a low-rank collapse. This suggests that the difficult regimes (e.g., PoseX-SD and PoseBusters) are unlikely to be explained by a trivial loss of embedding variability alone.

At the same time, both the t-SNE projections and the cluster statistics indicate weak alignment between the pretrained embedding geometry and oracle-defined solver regions. The projections form multi-lobed clouds rather than clean solver-separated clusters, which is expected because the embeddings are not trained to discriminate docking pipelines. Consistently, silhouette scores are negative across benchmarks (Table 8), indicating substantial overlap between solver-associated regions under this oracle colouring, with the poorest separability observed on PoseX-SD and PoseX-CD.

Centroid-distance heatmaps provide a complementary solver-level view. On MOAD-curated, centroid separations are relatively homogeneous, consistent with a compact embedding geometry in which solver centroids are not strongly isolated. On PoseX benchmarks, the matrices become denser with repeated block structure, suggesting that many solvers occupy overlapping regions of the embedding space, which is compatible with the high oracle diversity observed there. PoseBusters exhibits a different pattern in which a small number of solvers (notably EquiBind, and to a lesser extent Uni-Mol) form more distinct centroid blocks relative to the remaining methods.

Overall, these diagnostics do not support a collapse of the embedding feature spectrum, but they do suggest that pretrained embeddings provide limited solver-specific separability under several workflows. In regimes where oracle diversity is high and embedding separability is weak (Table 8), MolAS tends to default towards an SBS-like strategy, consistent with the selection-collapse patterns in the main text.

**Table 8:** Summary of embedding-space statistics and oracle-landscape properties across benchmarks

Dataset	Cluster tightness	Silhouette score	VBS score	VBS entropy
MOAD-curated	3.305	−0.036	0.867	2.395
PoseX + Astex	2.709	−0.143	0.956	4.699
PoseX-SD	2.960	−0.173	0.972	4.675
PoseX-CD	2.431	−0.183	0.944	4.590
PoseBusters	2.982	−0.047	0.763	3.205

Cluster tightness and Silhouette score quantify per-algorithm embedding dispersion and separability. VBS score and VBS entropy summarise the oracle success rate and the distribution of oracle-best algorithms

#### Top-k overlap across post-processing settings

Fig. 15 reports the top-*k* overlap curves for all three post-processing settings. Across settings, the qualitative pattern is consistent: overlap is typically low for small-to-moderate *k* (apart from some $k=1$ when the top solver coincides) and increases towards 1 as *k* approaches *m*, reflecting instability among near-top solvers. Accordingly, these curves primarily diagnose upper-tail hierarchy shifts across protocols, which are most relevant for oracle labels and algorithm-selection transfer.

## Abbreviations

AS: Algorithm selection
BCE: Binary cross-entropy
CD: Cross docking
EGNN: Equivariant graph neural network
GAT: Graph attention network
GINE: Graph isomorphism network
GNN: Graph neural network
ML: Machine learning
MM-min: Molecular-mechanics energy minimisation
MolAS: Molecular embedding-based algorithm selector
NDCG: Normalized discounted cumulative gain
PB: PoseBusters
PL: Pairwise logistic
RMSD: Root-mean-square deviation
SBS: Single best solver
SD: Self docking
VBS: Virtual best solver
